# Single catheter approach for treatment of premature ventricular contractions

**DOI:** 10.1002/clc.24250

**Published:** 2024-03-07

**Authors:** Patrick Dilk, Borislav Dinov, Angeliki Darma, Andreas Bollmann, Nikolas Dagres, Gerhard Hindricks, Arash Arya

**Affiliations:** ^1^ Department for Electrophysiology Heart Centre Leipzig Leipzig Germany; ^2^ Department of Cardiology and Angiology University Hospital of Giessen Giessen Germany; ^3^ Department for Electrophysiology Deutsches Herzzentrum der Charité Berlin Germany; ^4^ Department for Electrophysiology University Halle Halle Germany

**Keywords:** catheter ablation, premature ventricular contractions, ventricular arrhythmia

## Abstract

**Background:**

Catheter ablation has become one of the main treatment strategies in patients with premature ventricular complexes (PVC). The successful mapping and ablation can be performed with an ablation catheter without additional diagnostic catheters.

**Hypothesis:**

We hypothesize that using a single catheter for PVC ablation may decrease complications, procedure time, and fluoroscopy exposure while maintaining comparable success rates.

**Methods:**

Sixty‐nine consecutive patients with PVC were treated with a single catheter approach compared to a historical cohort, in which a conventional setup was used. Propensity score matching was conducted with a 1:1 ratio. Outcome parameters included acute procedural success with elimination of all premature ventricular contractions after catheter ablation, procedural data as well as complication rates.

**Results:**

Patients treated with a single catheter approach had shorter total procedure (60 minutes [IQR: 47,5–69,0 minutes] vs. 90 minutes [IQR 60–120 minutes]; *p* = 0.001) and fluoroscopy times (218 seconds [IQR: 110,5–446 seconds] vs. 310 seconds [IQR 190–640 seconds]; *p* = 0.012), which consecutively leads to a reduction of radiation exposure signified by a lower dose area product (155 cGycm² [IQR 74.4–334.5 cGycm²] vs. 368.4 cGycm² [IQR: 126–905.4 cGycm²]; *p* value 0.009). Acute procedural success rates were comparable in both groups (54 [84.3%] in the single catheter approach group and 58 [90.6%] in the conventional group; *p*: 0.287).

**Conclusion:**

A single catheter approach for the treatment of PVC is associated with a reduction of procedure‐ and fluoroscopy time, as well as a lesser radiation exposure, while maintaining equivalent acute success and complication rates compared with a conventionally used catheter setup.

## Graphical abstract

In our study, a total of 309 patients were initially treated for PVC ablation from January 2020 to February 2021. Following propensity score matching for a balanced comparison with a historical cohort (2016–2020), the final analysis included 128 patients, with 64 in each group. The investigation into the single catheter approach revealed a 33% reduction in procedure time, with a median of 60 minutes [IQR: 47.5–69.0 minutes] compared with 90 minutes [IQR 60–120 minutes] in the conventional group (*p* = 0.001). Acute success rates were comparable between groups, with 84.3% in the single catheter approach and 90.6% in the conventional group (*p* = 0.287). Additionally, a substantial 58% decrease in dose area product was observed in the single catheter approach, with a median of 155 cGycm² [IQR 74.4–334.5 cGycm²] compared with 368.4 cGycm² [IQR: 126–905.4 cGycm²] in the conventional group (*p* = 0.009). These outcomes underscore the procedural efficiency of the single catheter approach, marking a notable advancement in PVC ablation methodologies.

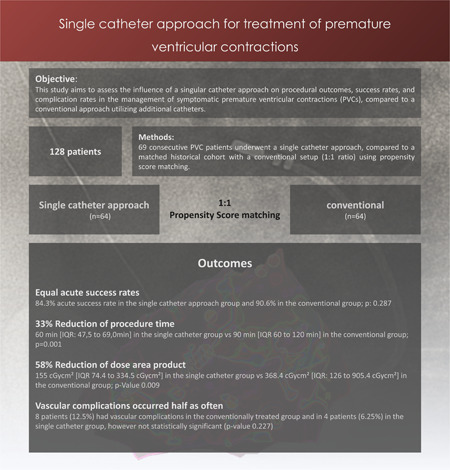

## 1 INTRODUCTION

Catheter ablation has become one of the main treatment strategies in patients with symptomatic palpitations caused by premature ventricular contractions (PVC), when pharmacological treatment is insufficient in suppression of PVC burden or not desired.^1^, ^2^ Furthermore, it has been shown to restore left ventricular function in patients with PVC induced cardiomyopathy,^3^, ^4^, ^5^ as well as decrease the burden of PVC induced episodes of ventricular fibrillation (VF).^6^ This has led to a class I recommendation in recent guidelines, when PVCs are considered to be idiopathic or LV dysfunction is present and class IIa in nonidiopathic PVCs.^2^

The success rate of ablation of PVC is considered to range from 84% to 92%,^7^, ^8^ while maintain a low intraprocedural complication rate ranging from 2.5% to 5%.^8^, ^9^, ^10^ However, acute success rates differ dependent on the localization of the PVC ranging from approximately 90% in patients with outflow tract PVCs to 70% in patients with an epicardial exit.^8^ But despite these promising acute procedural outcomes, long‐term reduction of PVC burden after catheter ablation can be improved in conjunction with additional antiarrhythmic drug therapy.^8^ Nonetheless, acute procedural success remains the most valuable predictor for long‐term success.^11^

Usually, the setup for ablation of PVC includes an atrial catheter which is placed in the coronary sinus (CS), a right ventricular catheter (RVA), a catheter which is placed alongside the bundle of His and a separate ablation catheter. However, successful ablation side of PVC mainly depends on prematurity of the PVC on the uni‐ and bipolar recording of the ablation catheter, as well as morphological aspects based on pace mapping.^12^, ^13^

It seems reasonable to reduce the amount of diagnostical catheters to only an ablation catheter, when the symptoms of a patient can be clearly related to PVC. We therefore hypothesize, that the utilization of a single catheter approach for ablation of PVC may reduce vascular complications, procedure, and fluoroscopy times, while maintaining the same acute success rate.

## 2 METHODS

### 2.1 Patients

From January 2020 to February 2021, patients with symptomatic PVC were treated with a single catheter approach, which is defined by solely using the ablation catheter for diagnostic pacing maneuvers and radiofrequency ablation (see below). Before the procedure, patients underwent diagnostic work up including eighter echocardiography or cardiac magnetic resonance tomography to define ventricular function and the presence of structural heart disease. If indicated, myocardial stress testing or coronary angiography was performed as part of the clinical routine. Ischemic heart disease was defined as history of myocardial infarction and presence of subendocardial scar in CMR imaging in combination with coronary artery disease; nonischemic cardiomyopathy was defined as impaired left ventricular function with a left ventricular ejection fraction <50% without coronary artery disease or presence of subendocardial scar, which can be attributed to a preceded myocardial infarction. Nonischemic cardiomyopathy was defined according to the criteria of the European Society of Cardiology Working Group on Myocardial and Pericardial Diseases.^14^ All patients provided written informed consent for the procedure in accordance with the institutional guidelines.

No patients were excluded or selected based on PVC location, ensuring a comprehensive evaluation of the single catheter approach across diverse cases in our study.

These patients were compared with historic cohort of patients underwent catheter ablation for PVC from 2016 to 2020 where a conventional electrophysiology study catheter setup, incorporating at least a quadripolar catheter, which was placed at the apex of the right ventricle (RVA) or additional diagnostic catheters were introduced. To minimize treatment selection bias as well as confounding, we emulated balance for both cohorts, determined by baseline characteristics utilizing a propensity score (see below). Ethic approval was granted by the Ethics Committee at the University of Leipzig (IRB‐128/17‐ek).

### 2.2 Catheter ablation of PVC

All patients underwent an electrophysiological study in the fasting state. Deep sedation could be performed at the discretion of the investigator (e.g., bradycardia associated PVC) using propofol, midazolam, and fentanyl. After vascular access and introduction of arterial/venous sheaths the diagnostical catheters and/or ablation catheters were placed. The initial preferred vascular access was chosen primarily by the morphology of the PVC based on the 12‐lead electrocardiogram and on the discretion of the investigator.

In the conventional group, at least a quadripolar catheter was placed in the right ventricular apex and/or at the bundle of his, as well as a decapolar catheter into the CS under fluoroscopic guidance. In the single catheter group, only the mapping/ablation catheter was introduced. In case of PVC originating from the left ventricle, which cannot be assessed by a retrograde access via the aorta, a single transseptal puncture was performed fluoroscopically in combination with a steerable sheath (Agilis; Abbott). Intravenous heparin was administered before the transseptal puncture or retrograde access, maintaining an activated clotting time of 250–350 seconds.

Electroanatomic mapping was performed using the CARTO‐3 system (Biosense Webster) or the EnSite‐X Navigation system (Abbott). Signals were continuously recorded using a multichannel recording system (Prucka CardioLab; GE Healthcare). The targeted location for ablation of PVCs was defined by morphology criteria utilizing pace mapping, as well as earliest local activation time, measured by a bipolar electrogram preceding the surface QRS by more than 25 milliseconds, in conjunction with an QS unipolar electrogram configuration on the distal electrode. In case of suspected LV Summit PVCs, the CS Catheter was advanced deep into the CS. In the conventional group, signals were annotated with the RVA catheter as reference, while in the single catheter group annotation was done according to the surface ECG.

In both the single catheter and conventional approaches, identical ablation catheters (F‐Type, irrigated tip, Thermocool, Biosense Webster; Flexability SE™ Ablation Catheter, Abbott) were utilized. Radiofrequency alternating current was applied in a unipolar mode with specific parameters: an upper‐temperature limit of 42°C, maximum power of 50 W, and irrigation flow ranging from 15 to 30 mL/min.

After cessation of PVCs, additional isoprenaline was administered to verify if the clinically relevant PVC do reoccur under beta‐adrenergic stimulation. The procedure was defined as complete successful, when no PVC occurred within 30 minutes; partially successful when there was a quantitative reduction of the predominant PVC morphology or additional PVC morphologies occurred apart from the clinically relevant. Vascular complications included postinterventional hematoma, arterio‐venous fistula, pseudoaneurysm, and active bleeding.

### 2.3 Statistical analysis

Categorial variables are summarized by count and percentage and compared with the use of the *χ* ^2^ test. Continuous variables are described as median and interquartile range (IQR) and compared with the Mann–Whitney *U* test. A two‐sided *p* value of less than 0.05 was considered to indicate statistical significance. To assess clinically relevant differences between patients treated with a single catheter approach compared with conventional treatment, we used a propensity score matching analysis, including relevant baseline characteristics, incorporating demographic characteristics (Age, gender, BMI); pre‐existing comorbidities (e.g., arterial hypertension, diabetes, coronary artery disease etc.), renal function, as well as PVC origin and vascular access—all matching variables are represented in Table 1. Propensity Score matching was performed using the “nearest‐neighbor approach,” with a 1:1 ratio within a caliper width of 0.2 of the standard deviation of the logit of the propensity score. We used the matching package from the R Software, version 3.15 for propensity score matching within SPSS statistical package version 22 (IBM).

**Table 1:** Baseline characteristics.

	Single catheter		Conventional		*p* Value
	*n* = 64		*n* = 64		
Age (years)	63	(47.5–69.0)	61	(48.5–71.5)	0.922
Female Gender	20	31.25%	21	32.81%	0.85
BMI (kg/m²)	26,9	(24.4–32.4)	28,1	(24.2–30.9)	0.834
Arterial hypertension	38	59.38%	40	62.50%	0.718
Diabetes mellitus	10	15.63%	11	17.19%	0.812
Coronary artery disease	15	23.44%	18	28.13%	0.546
Prior cardiac surgery	5	7.81%	5	7.81%	1
Atrial fibrillation	11	17.19%	13	20.31%	0.652
CRT‐D	2	3.13%	3	4.69%	0.529
One chamber ICD	3	4.69%	2	3.13%	0.529
LVEF (%)	54,8	(45.2–59.9)	50,2	(43.5–58.8)	0.397
Cardiomyopathy					
Idiopathic	39	60.94%	38	59.38%	0.857
Ischemic	5	7.81%	7	10.94%	0.546
Nonischemic	20	31.25%	19	29.69%	0.848
GFR (mL/min)	78	(66.0–99.5)	81	(65.0–96.0)	0.623
β‐Blocker	42	65.63%	43	67.19%	0.852
Type I AAD	1	1.56%	2	3.13%	0.561
Type III AAD	1	1.56%	0	(0.0–0.0)	0.317
PVC burden (PVC/d)	20 000	(11 176–32 472)	21 492	(10 300–29 000)	0.948
Localization					
AMC	4	6.25%	3	4.69%	0.699
LV free wall	6	9.38%	6	9.38%	1
LV summit	2	3.13%	3	4.69%	0.65
LVOT	33	51.56%	33	51.56%	1
Papillary muscle	0	(0.0–0.0)	1	1.56%	0.317
RV free wall	4	6.25%	3	4.69%	0.699
RVOT	15	23.44%	15	23.44%	1
Pleomorphic PVC	20	31.25%	17	26.56%	0.56
Access to PVC					
Antegrade	27	42.18%	25	39.06%	0.846
Retrograde	37	57.81%	39	60.09%	0.846
Transseptal	8	12.50%	7	10.94%	0.784

Abbreviations: AAD, antiarrhythmic drug; AMC, aorto‐mitral continuity; BMI, body mass index; CRT‐D, cardiac resynchronization therapy defibrillator; GFR, glomerular filtration rate; ICD, implantable cardioverter defibrillator; LV, left ventricular; LVEF, left ventricular ejection fraction; LVOT, left ventricular outflow tract; PVC, premature ventricular contraction; RV, right ventricular; RVOT, right ventricular outflow tract.

## 3 RESULTS

### 3.1 Baseline characteristics

**Figure 1:**
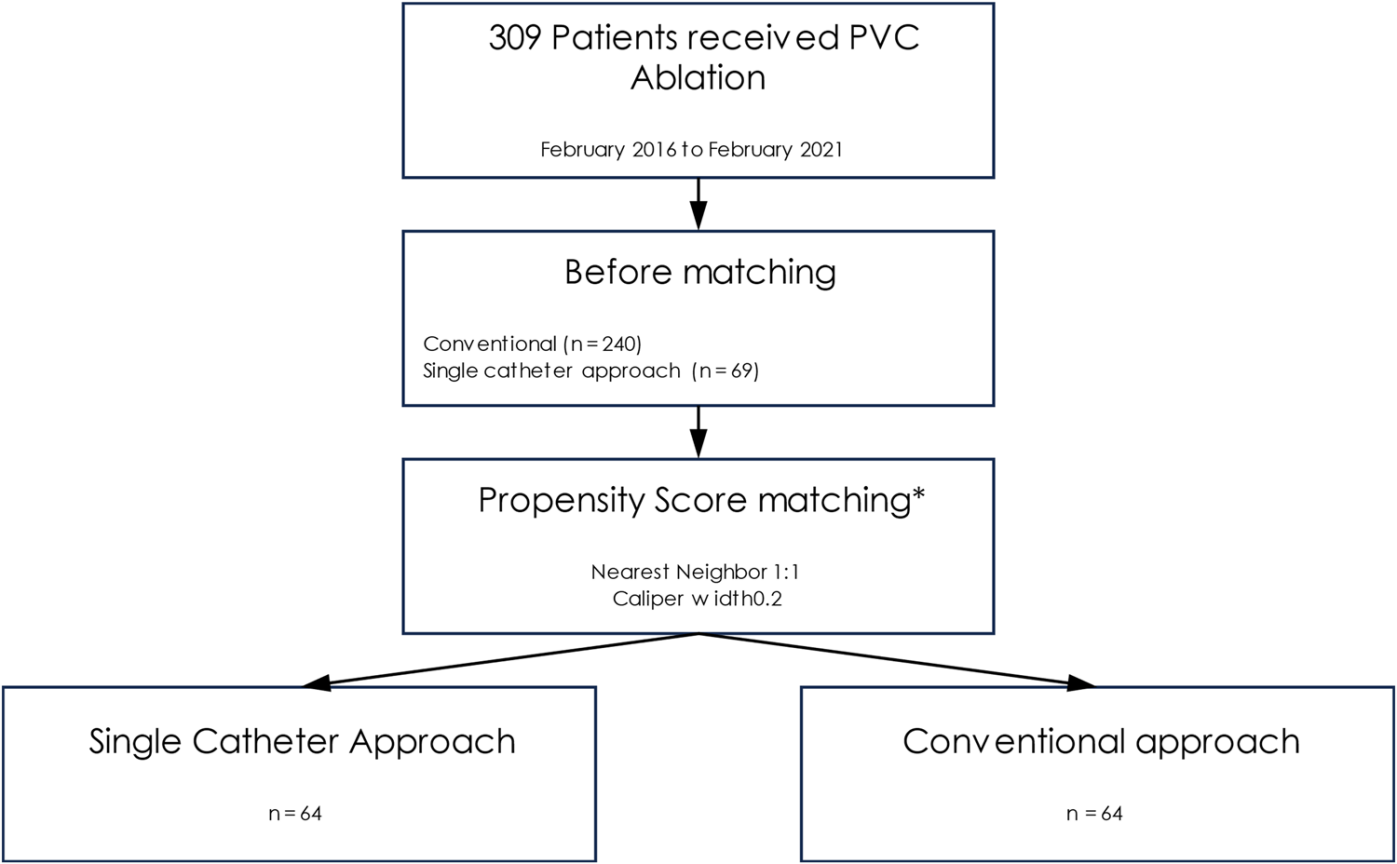
Study flowchart. *Patients were matched for the following covariates: age, gender, BMI, arterial hypertension, diabetes mellitus, coronary artery disease, prior cardiac surgery, CRT‐D, LVEF, type of cardiomyopathy, GFR, antiarrhythmic medication, PVC burden, localization of PVCs and pleomorphic PVCs. BMI, body mass Index; CRT‐D, cardiac resynchronization therapy defibrillator; GFR, glomerular filtration rate; LVEF, left ventricular ejection fraction; PVC, premature ventricular contraction.

From February 2016 to February 2021 a total of 309 patients were treated with catheter ablation for PVCs. Sixty‐nine of these were treated with a single catheter approach (22%), only using one ablation catheter for the whole procedure. After propensity score matching a total of 128 patients were included in the final analysis; 64 patients in each treatment arms (Figure 1). All baseline characteristics were balanced after propensity score matching (Table 1). The median age of the patients was 60.5 years (IQR 48–70), 41 patients (32%) were female. The most frequent origin of PVC included the left (51.5%) and right (23.4%) ventricular outflow tract. Most PVC were considered to be idiopathic (60.1%).

### 3.2 Procedural outcomes

Patients treated with a single catheter approach had significant shorter total procedure times compared with a conventional approach (median 60 minutes, IQR 50–87.5 minutes, vs. 90 minutes in the conventional arm, IQR 60–120 minutes; *p* value = 0.001), as well as shorter fluoroscopy times (median 218 seconds, IQR 110.5–446 seconds, vs. 310 seconds in the conventional arm, IQR 190.5–640 seconds; *p* value 0.012), correlating with a reduction of the applied dose area product (median 368.4 cGycm², IQR 126–905.4 cGycm², vs. 155 cGycm² in the conventional arm, IQR 74.4–334.5 cGycm²; *p* value 0.009).

No significant differences were observed among acute success rates after catheter ablation. Complete elimination of PVC was achieved in 58 patients (90.6%) in the conventional arm and in 54 patients (84.3%) in the single catheter approach arm (*p* value 0.287; Table 2).

**Table 2:** Procedural outcomes.

	Single catheter		Conventional		*p* Value
	*n* = 64		*n* = 64		
Procedure time (min)	60	(50.0–87.5)	90	(60.0–120.0)	0.001
Fluoroscopy time (s)	218	(110.5–446.0)	310	(190.5–640.0)	0.012
Dose area product (cGycm²)	155.1	(74.4–334.5)	368,4	(126.0–905.4)	0.009
Nr. venous punctures	1	(0.0–1.0)	2	(2.0–3.0)	<0.001
Nr. arterial punctures	1	(0.5–1.0)	1	(1.0–1.0)	0.146
Nr. total punctures	1	(1.0–2.0)	3	(2.0–4.0)	<0.001
Nr. ablations points	6.5	(3.0–17,5)	10	(4.0–16.0)	0.356
Ablation time	301	(132.5–752.5)	355.5	(187.0–783.5)	0.213
Acute success					
Partially	9	14.06%	3	4.69%	0.078
Complete	54	84.38%	58	90.63%	0.287

### 3.3 Complications

Pericardial effusion occurred in two patients (3.13%) in the conventional arm and in one patient (1.56%) where a single catheter approach was used (*p* value 0.561). Vascular complications occurred in eight patients (12.5%) in the conventionally treated group and with four patients (6.25%) half as often in the single catheter group, however not statistically significant (*p* value 0.227).

## 4 DISCUSSION

This study was designed to determine whether the use of a single catheter approach would have an effect on procedural outcomes, success rates, and complications rates in the treatment of PVCs. According to our study, in patients with symptomatic PVC, limiting the number of diagnostic catheters to only one ablation catheter resulted in significant reductions in the total procedure time, fluoroscopy time, and dose area product as well, while maintaining comparable success rates and complications when compared with conventional catheter setups for electrophysiological studies. Differences in “partial success” rates between single catheter and conventional methods may stem from procedural nuances, not method superiority. The subjectivity of “partial success” criteria, considering PVC characteristics, requires cautious interpretation. Both methods, however, showed comparable success rates in eliminating all PVCs, highlighting the need for careful consideration of procedural complexities and patient‐specific factors.

No differences were observed regarding procedure‐associated complications in both groups. Efforts to minimize vascular complications in both groups included strict adherence to established protocols, meticulous catheter placement, and routine ultrasound‐guided vascular access. Pericardial effusion instances were primarily associated with ablation procedures or complex septal punctures rather than the presence of additional catheters. Notably, there was no observed damage to the cardiac conduction system in either group.

Palpitations, or worsening left ventricular function in patients with heart failure symptoms, may not necessarily require additional electrophysiological testing, when the symptoms are attributed to a substantial burden of PVCs on continuous rhythm monitoring. To localize these PVCs, a variety of algorithms are available, including the identification of potential epicardial exit points and catheter ablation sites.^15^, ^16^, ^17^ The ideal ablation site for PVCs is characterized by a similar 12‐lead‐ECG morphology when applying pace mapping, as well as a sufficient duration of local activation on the ablation catheter before the onset of surface QRS of at least 25 milliseconds in conjunction with a QS configuration on a unipolar recording of the distal ablation catheter electrode.^12^, ^13^ Using only one catheter, the latter can be used to perform both mapping and ablation without the need for additional diagnostic catheters.

Sousa et al. conducted a study to determine whether using the PentaRay catheter for activation mapping in PVC ablation procedures could lead to shorter procedure times. They divided 136 consecutive patients into two groups: the Study group, where the PentaRay catheter was used, and the Control group, where the ablation catheter was used. The Study Group had significantly shorter procedure times (110 ± 33 minutes) compared with the Control Group (134 ± 50 minutes, *p* < 0.01). Although there were no significant differences in acute success rates (95.6% in the Study Group vs. 90.1% in the Control Group, *p* = 0.49), the use of the PentaRay catheter resulted in notable time savings during the PVC ablation procedures. However, it is important to note that the procedure times observed in this study were longer than the procedure times achieved with our investigation utilizing a single catheter approach.^18^

The study of Dukkipati et al. evaluated the efficacy and safety of infusion needle ablation (INA) for treating refractory PVC. INA involved using a catheter with an extendable/retractable needle to perform targeted ablation. Among 35 patients who underwent INA, acute PVC elimination was achieved in 71.4% of cases. The procedure had a reasonable safety profile, with adverse events including heart block, femoral artery dissection, and pericardial effusions occurring in 14.3% of patients, all of which were successfully managed percutaneously. In terms of procedural parameters, the study reported a mean fluoroscopy time of 27.9 minutes and a mean procedure time of 293.7 minutes, indicating the overall duration of the INA procedure. As mentioned above, this trial had also higher Procedure and fluoroscopy times than the single catheter approach we investigated.^19^

Jáuregui et al. aimed to assess the benefits of an automatic LAT acquisition protocol using wavefront annotation and an ECG pattern matching algorithm during PVC ablation procedures. One hundred consecutive patients were enrolled and randomized to either the automatic (AUT) or manual (MAN) annotation protocols. The results showed that mapping and procedure times were significantly shorter in the AUT group compared with the MAN group (25.5 ± 14.3 minutes vs. 32.8 ± 12.6 minutes, *p* = 0.009, and 54.8 ± 24.8 minutes vs. 67.4 ± 25.2 minutes, *p* = 0.014, respectively), the latter being comparable to our investigation. However, success rates were similar between the two groups. Clinical success was significantly better in the AUT arm (100% vs. 88%; *p* = 0.01), but not during follow‐up (92% AUT vs. 88% MAN; *p* = 0.51). No procedure‐related complications were reported.^20^

The success of future outpatient‐based ablation treatments will be heavily influenced by the ability to minimize the incidence of complications (e.g., by reducing procedure times and vascular punctures), making it a crucial area of focus for healthcare providers. As a result, we chose not to use advanced multielectrode catheters for activation mapping since these complex devices cannot provide the simplicity of a single catheter approach. In this context, a single catheter approach may provide a safe and feasible treatment option for PVC, especially those that are of idiopathic origin.

While our study supports the efficiency of the single catheter approach in PVC ablation, caution is advised in cases with para‐hisian PVCs due to their proximity to the cardiac conduction system. The potential risk of inducing complete heart block in this scenario warrants prudence. For such cases, a traditional approach with multiple catheters, especially for ventricular backup pacing, may be more prudent. The decision on the approach should be thoughtful, considering individual factors and anatomical considerations.

This investigation had several limitations: (1) this was a retrospective, monocentric analysis and not prospectively randomized or multicentric. (2) The propensity matched analysis was performed with a 1:1 ratio—a higher ratio, for example, 1:2 would be more appropriate for this kind of analysis, but was limited by the number of matched baseline characteristics, mainly PVC origin. (3) Despite recognizing the correlation between acute and long‐term success, the absence of data on recurrence rates, PVC burden postablation, symptom alleviation, and medication needs was notable due to follow‐ups conducted in an ambulatory, out‐of‐hospital setting. We acknowledge these limitations and suggests a need for enhanced follow‐up measures, proposing a direct, truly randomized trial with continuous rhythm for more conclusive insights into the single catheter approach's efficacy and sustainability.

In conclusion, in a comparison of a conventional catheter setup, compared with a limitation to solely an ablation catheter, which is used for diagnostic and therapeutic maneuvers, for the interventional treatment of PVC, we found that a single catheter approach is associated with a reduction of procedure‐ and fluoroscopy time, as well as a lesser radiation exposure surrogated by dose area product, while maintaining equivalent acute success and complication rates.

## CONFLICT OF INTEREST STATEMENT

The authors declare no conflict of interest.

## Data Availability

The data that support the findings of this study are available from the corresponding author upon reasonable request.
